# Adenovirus serotype 7 associated with a severe lower respiratory tract disease outbreak in infants in Shaanxi Province, China

**DOI:** 10.1186/1743-422X-8-23

**Published:** 2011-01-18

**Authors:** Liuying Tang, Li Wang, Xiaojuan Tan, Wenbo Xu

**Affiliations:** 1 National Institute for Viral Disease Control and Prevention, Chinese Center for Disease Control and Prevention. State Key Laboratory for Molecular Virology & Genetic Engineering. 27, Nanwei Road, Room 507, Xuanwu District, Beijing, 100050, P. R. China; 2 Shaanxi Center for Disease Control and Prevention, Xi'an, P. R. China

## Abstract

**Background:**

Pneumonia caused by adenovirus infection is usually severe especially with adenovirus serotype 7 commonly associated with lower respiratory tract disease outbreaks. We reported an outbreak of 70 cases of severe pneumonia with one death of infants in Shaanxi Province, China. Sampling showed adenovirus 7 (Ad7) as the primary pathogen with some co-infections.

**Results:**

Two strains of adenovirus and two strains of enterovirus were isolated, the 21 pharynx swabs showed 14 positive amplifications for adenovirus; three co-infections with respiratory syncytial virus, two positive for rhinovirus, one positive for parainfluenza 3, and four negative. Adenovirus typing showed nine of the nine adenovirus positive samples were HAdV-7, three were HAdV-3 and two were too weak to perform sequencing. The entire hexon gene of adenovirus was sequenced and analyzed for the two adenovirus serotype 7 isolates, showing the nucleic acid homology was 99.8% between the two strains and 99.5% compared to the reference strain HAdV-7 (GenBank accession number AY769946). For the 21 acute phase serum samples from the 21 patients, six samples had positives results for ELISA detection of HAdV IgA, and the neutralization titers of the convalescent-phase samples were four times higher than those of the acute-phase samples in nine pairs.

**Conclusions:**

We concluded adenovirus was the viral pathogen, primarily HAdV-7, with some co-infections responsible for the outbreak. This is the first report of an infant pneumonia outbreak caused by adenovirus serotype 7 in Shaanxi Province, China.

## Background

Human adenoviruses cause a wide spectrum of diseases. Pneumonia caused by adenovirus infection is usually severe especially with adenovirus serotype 7 commonly associated with lower respiratory tract disease outbreaks. During the last global survey, approximately one-fifth of all HAdV infections reported to the World Health Organization (WHO) were attributed to HAdV-7[1,2], the diseases reported include respiratory tract illnesses and conjunctivitis. In infants and immuno-compromised populations, HAdV-7 can cause outbreaks of severe disease; and in a few cases can lead to death[3]. Multiple HAdV-7 genome types have been identified using restriction enzyme analyses[4]. Global prevalence patterns of these HAdV-7 genome types shift over time[1,5]. Reported cases of adenovirus infection have increased in China recently where most of the outbreaks are caused by adenovirus 3 and one had HAdV-11; and the infected groups were primary and middle school students[6-8]. Here we report an outbreak that affected in young children of Xixiang County of Shaanxi Province, China. Clinical specimens were collected from the admitted patients and we performed pathogen detection and analysis. This showed adenovirus serotype 7 was the primary viral pathogen with some co-infection responsible for the infant pneumonia mortality. This is the first report in ten years of an outbreak of infant pneumonia caused by HAdV-7 in China, and the first report ever from Shaanxi Province.

## Results

### Outbreak characteristics

**Figure 1:**
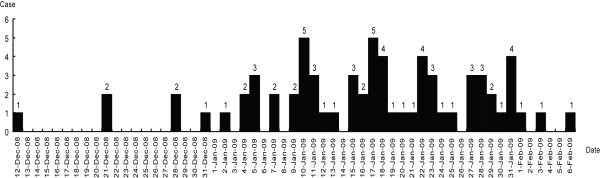
**The distribution of the 70 cases during the infant pneumonia outbreak**. On 8 December 2008, the first case was observed at the Xixiang Hospital, Shaanxi Province who presented febrile symptoms. The number of similar cases increased dramatically by 9 January. By 9 February 2009, the outbreak affected a total of 70 children in the Hanzhong area. These cases were identified based on a case definition and by conducting an active epidemiology search.

The disease was suspected to be of "unknown origin pneumonia" at the beginning of the outbreak; and quickly SARS and/or avian influenza were precluded with SARS-coronavirus and H5N1 specific detection, bacterium infection was precluded as well. The first case was reported on 15, cases accumulated in a short period and peaked on 17 Jan 2009 (Figure 1). Case epidemiology proceeded for 70 patients (32 reported using the internet reporting system directly and 38 during an active investigation from the four hospitals in Hanzhong). The age of the patients was from 40 days to 9 years; primarily in the 0-3 year range. Endemic distribution was scattered in some villages with the most in Xixiang County without a central tendency. Among the 70 patients, the admitting diagnosis was 56 with bronchopneumonia, 11 with bronchitis, two with acute tonsillitis, and one with lobar pneumonia. Clinical manifestations included fever (84.5%) with the highest at 40.5°C and a median of 38.8°C; and most cases presented with cough and some with asthma.

### PCR or RT-PCR

PCR or RT-PCR was performed with five pharynx swabs specimens collected from the first reported pneumonia patients using primer sets specific for respiratory viruses with the Seeplex RV Detection Set I. The results showed all of the five specimens were positive for human adenovirus and included a further 16 pharynx swabs for a total of twenty-one pharynx swabs: 14 were positive for adenovirus, three had a co-infection with respiratory syncytial virus, two positive for rhinovirus, one positive for parainfluenza 3, and four were negative. Of the 14 adenovirus positive samples, partial hexon gene sequencing results showed the 12 specimens were species B of HAdV, nine HAdV-7 and three HAdV-3 and amplification bands of two specimens were too weak to perform sequencing.

### Virus isolation

All 21 clinical specimens were separately inoculated into HEp-2 cells, when cell pathogenic effection (CPE) was observed, it occurred within three passages after inoculation in all cases. A characteristic adenovirus-like CPE was observed in the HEp-2 cells from two pharynx swabs samples and an enterovirus-like CPE was found for the two other samples.

### Molecular analysis of the two HAdV isolates

**Figure 2:**
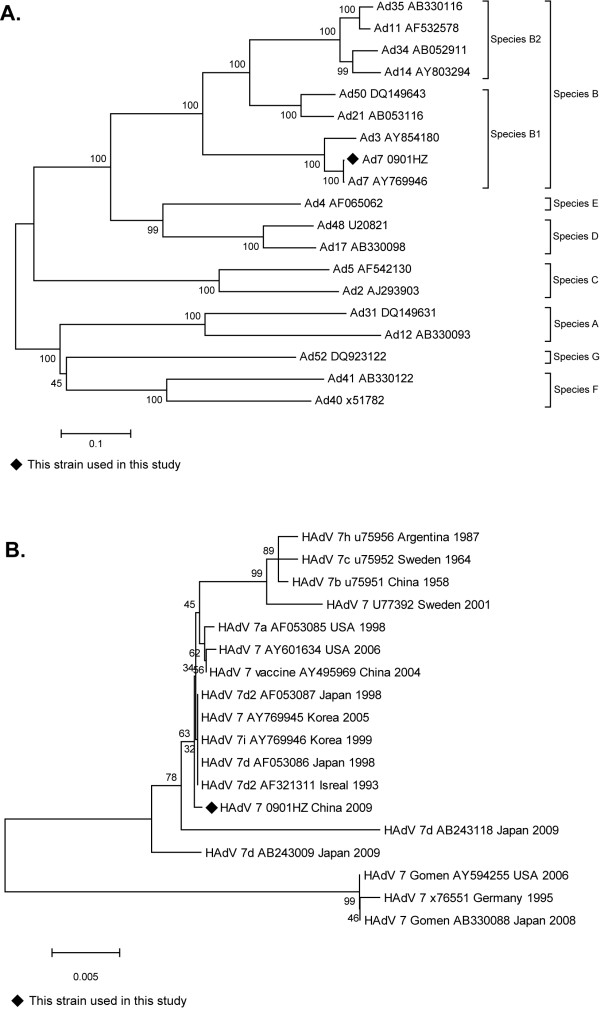
**Phylogenetic analysis of the entire hexon gene for strain Ad7 0901 HZ described in this study and other reference strains of adenovirus**. The phylogenetic tree generated using the neighbor-joining method. Bootstrap values are provided at the basal nodes of each species (species A to G). (A) Strain 0901 HZ was identified as a HAdV-7 strain belonging to the B1 species; (B) The phylogenetic tree of strain 0901 HZ compared to other HAdV-7 reference strains.

The entire hexon genes were amplified from the two adenovirus isolates using PCR with adenovirus-specific primers to obtain the predicted product of 3,162 bp (Table 1). Sequence determination showed the two viral isolates had 99.8% homology comparing their nucleic acid sequences. A viral strain designated HAdV7-0901 HZ was isolated from the pharynx sample of the dead patient; and the strain was used for phylogenetic analysis (Figure 2). The coding sequence for the HAdV-7 0901 HZ hexon was 2,805 bp, 96.3-99.8% with HAdV-7 prototype and vaccine strains comparing their nucleic acid sequences (AY594255, AY769946). Where the hexon is a 935 amino acid protein, sharing 95% amino acid identity with its homolog (HAdV-7, reference AY769946). The detection of respiratory syncytial virus, rhinovirus, parainfluenza virus and enterovirus were confirmed by sequencing (data not shown).

**Table 1:** Primers sequences used sequencing analysis of the adenovirus hexon gene

primers	Sequence (5'-3')	position	amplicon length(bp)
1U	GAACAGCATCGTGGGTCT	18186-18203	499
1L	GGACCTCTATCAAGCAC	18668-18684	
2U	CGGGAGGACAATACATAC	18569-18586	512
2L	CCTTCGGTTGGTGTTACT	19063-19080	
3U	AGCCTCAAGTTGGAGAAGA	18909-18927	522
3L	GCAAAAGCTGATATGACAG	19412-19430	
4U	CATTGGCTTCAGGGATAAC	19288-19306	478
4L	TGGCGTGTACTTGTAAAC	19748-19765	
5U	GGCAACAATCTGGCTATG	19661-19678	493
5L	GAGGTTGATGCTGGTGAA	20136-20153	
6U	TGGAAATGACCTCAGAAC	20089-20106	515
6L	GAACCAGGAACCAGTCTT	20586-20603	
7U	GTGGATGGGGAAGGATAC	20543-20560	506
7L	TAAAGCAGGGTGGGCTCA	21031-21048	
8U	CATACCGTTCTCCAGCAACT	20914-20933	509
8L	ATCAAAAAGGTAGCAGGT	21405-21422	
9U	CGCCATAGTCAACACTGC	21330-21347	486
9L	TATCCATACGGTCAAACG	21798-21815	

### Serology assays

The ELISA for HAdV IgA was performed using the 21 serum samples from the acute phase collected from 21 patients. Six samples had positives and three suspension positive (which the OD value is closed to the cut-off) results for IgA. We used the entire virion of the HAdV-7 strain isolated and used the identified strain HAdV7-HZ0901 as the neutralization virus. The CCID_50 _was determined to be approximately 10^5.0 ^CCID_50s_/50 μ.

Conventional neutralization tests were performed using 12 pairs of paired serum samples. We found the neutralization titers of the convalescent-phase samples were four times higher than those of the acute-phase samples in nine pairs (Table 1). We noticed that there was not a good correlation between detection of IgA and fourfold rise in neutralizing antibody titer as neutralization test detection relies mainly on IgG rather than IgM or IgA, it is quite possible that during the early onset period of the disease as the serum specimen is collected, IgG has not appeared for some cases.

## Discussion

In the epidemiology investigation of this event, we observed more cases of upper respiratory tract infectious disease occurred from 1 January to 6 February in 2009 (389 cases) than in 2008 (261 cases), an increase of 48.8%, from the Xixiang Chinese Medicine Hospital and Hanzhong Central Hospital (unpublished data). We found climate factors changed such as drought showed little rain and lower temperature in the same season than previous years in this area. Other virus infections, such as human respiratory syncytial virus, human rhinovirus and seasonal influenza virus may contribute to the outbreak for the other forty-nine patients whose clinical specimens were unavailable for pathogen detection. In addition, nosocomial infection can not be precluded during the outbreak. This is because six of 21 patients visited the same hospital at almost the same time after the index case. As prolonged shedding of adenovirus and its hardy nature make it an ideal agent for nosocomial transmission, nucleotide sequence comparison strongly suggested that all six patients have the same strain of adenovirus in their pharynx swabs give strong information for the nosocomial transmission of infection. A surveillance network for adenovirus infection has not been established; and adenovirus vaccines are presently unavailable in China. Most of the adenovirus infections especially severe pneumonia in infants was diagnosed clinically without laboratory confirmation, especially in county hospitals. Additionally, no HAdV-7 strains have been isolated and no population immunity survey has been reported from the Hanzhong areas. In the outbreak, there was no close correlation among most of the patients where they presented a diffused distribution and with higher occurrence in Xixiang County of the Hanzhong area. The parents of the infants denied having contact histories with similar patients or any history of travel. Therefore, it is difficult to determine the adenovirus origin for the outbreak.

HAdV-7 has multiple genome types, in the early 1980s, a new genome type Ad7d became the prevalent dominant strain[9]. Ad7d was isolated only in China from 1958-1984 and was dominant during 1980-1994. It was the representative genome type in Asian nations until 1998[10]. In a long-term survey of adenoviral pneumonia in Beijing (1958-1990), HAdV-7 was associated with a higher fatality rate than HAdV-3[11]. In Taiwan from 1980 to 2001, Ad7 and Ad4 were two emerging viruses, Ad7b was the predominant genotype of Ad7[12], while in some provinces of mainland China, such as Jiangsu, Hubei and Jiangxi, most of isolates from respiratory diseases outbreaks were Ad3[7,8,13]. Outbreaks of adenovirus serotype 7 infection have not been reported in China during the previous ten years; whereas a sporadic case of HAdV-7 infection has been reported in Beijing recently[14], and In 2002, Erdman et al. reported two emergent genome types of adenovirus type 7; both genome types were associated with epidemics, severe illness, and deaths outside of the United States[1]. There was a wide outbreak of adenovirus infection with five dead in Japan in 1995[15]. Then in 1998, the first report of an adenovirus 7d2 infection outbreak occurred in a pediatric chronic-care facility and tertiary-care hospital in Chicago with 67 infected and eight dead[16].

Although genome typing of the adenovirus serotype 7 isolates in this study has not been performed because reference strains were unavailable, a comparison with the available entire hexon gene sequences from the GenBank shows strain HAdV-7 0901 HZ isolated from the outbreak has the highest homology with HAdV7d2 from Israel, a 1993 isolate, HAdV-7d from Japan in 1998 and HAdV-7i from Korea in 1999 (GenBank accession number AF321311, AF053086 and AY769946, respectively) (Figure 2B). Comparison of the predicted amino acid sequences with other adenovirus 7 genotypes shows strain 0901 HZ lost glutamine at site 253 similar to the Korean strain; and at site 495, arginine took the place of serine[17]. The role of these changes in the adenovirus antigenicity is not known and requires further study. An adenovirus infection surveillance programme is going conducted in five provinces of China, including Shaanxi province, which will be helpful for chasing the transmission origin and more molecular epidemiology baseline data establishment in China.

## Conclusions

In the outbreak of the Shaanxi infant pneumonia, 56 cases were diagnosed with bronchopneumonia. Of the 21 pharynx swabs taken, 14 cases were shown to be positive for adenovirus; six cases were positive for adenovirus antibody with ELISA-IgA detection in the 21 acute phase serum samples; while the neutralization titers of the convalescent-phase samples were four times higher than those of the acute-phase samples for nine pairs. These results showed that adenovirus (primarily HAdV-7) was the primary pathogen in the outbreak. This is the first report of an infant pneumonia outbreak caused by adenovirus 7 in Shaanxi Province, China.

It is necessary to enhance the surveillance for a quick diagnosis of adenovirus infections for a nation-wide response to this emergency and re-emergent disease.

## Patients and Methods

The index case was a one-year-old female from Xixiang County, Hanzhong, Shaanxi Province. She had an onset on 15 January with an admission to Hanzhong Central Hospital with complaints of cough, expectoration asthma with a fever for 5 days. The clinical diagnosis was acute bronchitis with heart failure and toxic encephalopathy where the patient died on 30 January. Thirty-two cases of severe acute lower respiratory tract infections were reported through 9 February 2009; and another thirty-eight cases were found in four hospitals in Hanzhong city of Shaanxi Province with the definition of severe pneumonia:

1. Prolonged fever continuing at 37.5°C - 40°C.

2. Iconographic pneumonia with apparent respiratory symptoms.

3. Normal or lower total WBC.

4. No apparent improvement or became more severe after 3-day treatment with antibiotics.

### Specimen collection

During the outbreak of the disease, 21 pharynx swab specimens and 21 acute-phase sera samples were collected from 21 patients; and 12 returning patients gave convalescent-phase sera. The pharynx swab specimens were collected and transferred to 1 ml viral transport medium.

### Cell culture and virus isolation

The 21 pharynx swab specimens collected from the patients were inoculated onto HEp-2 cells and were cultured in a maintenance medium (Minimal Essential Medium containing 2% fetal calf serum, 100 U/ml penicillin G and 100 μg/ml streptomycin) at 37°C in a closed system without added CO_2_. Cultures exhibiting an adenovirus-like CPE were passed again to confirm the presence of the virus. Primary identification of positive isolates was performed using PCR with adenovirus-specific primers.

### Neutralization test

The stored serum samples were inactivated at 56°C for 30 min; diluted eight times with the maintenance medium; and filtered through a 0.22 μm filter membrane. Dilutions of the serum samples (1:8 to 1:1,024) were prepared and 50 μl of each dilution was added to four wells of a 96-well microplate. The entire virion of the HAdV strain isolated and identified was used as the neutralization virus. The 50% cell culture infective dose (CCID50) per 50 μl was calculated using the formula of Kärber[18].

### Elisa

An ELISA Classic adenovirus IgA kit (Institute Virion/Serion GmbH, W.rzburg, Germany) that enables the detection of serum antibodies against all serotypes of HAdV pathogenic for humans was used to detect HAdV immunoglobulin A (IgA) from the 21 acute phase sera samples from 21 patients.

### Extraction of viral nucleic acid and RT-PCR or PCR

The viral nucleic acid was directly extracted from the clinical specimens using a QIAamp mini-viral RNA extraction kit or a QIAamp DNA mini kit (Qiagen, Valencia, CA). Reverse transcription-PCR (RT-PCR) or PCR was performed using the Seeplex RV Detection Set I (RV6C00Y, Seegene, USA) that is designed to detect 11 types of RNA viruses and one type of DNA virus responsible for most respiratory diseases. The 11 RNA respiratory viruses include influenza A and B virus, human respiratory syncytial virus A and B, human metapneumovirus, human parainfluenzavirus[1,9,17], human rhinovirus A, and human coronavirus 229E/NL63 and OC43. The DNA respiratory virus was human adenovirus[19]. We also used the adenovirus species-specific PCR that can distinguish the six species of adenovirus and type-specific PCR of species B described by Pring-.kerblom[20]. The PCR was performed using primer pairs ADSD/AD52 as described by Zhen[6]. The PCR reaction was performed using a GeneAmp 9700 thermal cycler (Applied Biosystems). The amplification products were analyzed by electrophoresis of the samples in 1% agarose gels; and they were visualized with ethidium bromide under UV light.

### Sequence analysis

The PCR products were sequenced directly after purification (QIA gel extraction kit; Qiagen, KK, Japan) using the dye terminator method (Big Dye Terminator, version3.1, cycle sequencing kit; Applied Biosystems) with an ABI Prism 3100 genetic analyzer (Applied Biosystems). The primers of adenovirus used for sequencing are shown in Table 2. The primers of human respiratory syncytial virus, human parainfluenzavirus, human rhinovirus and enterovirus were donated by colleagues in other laboratories in the Institute.

**Table 2:** Patients information and the results for 21 pharynx swabs and paired sera analysis

ID code	gender	age	onset date	clinical diagnosis	multiplex PCR	adenovirus type	virus isolation	Adenovirus ELISA- IgA	Adenovirus nutralization antibody titer	
									
									Acute sera	convalescence sera
1	male	1y	31/01/2009	bronchopneumonia	+	HAdV-7	-	+	1:32	1:128
2	male	2y	31/01/2009	bronchopneumonia	+^a^	HAdV-3	-	-	1:128	1:128
3	female	2y	31/01/2009	bronchopneumonia	+	HAdV-3	-	+	<1:8	1:128
4	male	2y	09/01/2009	bronchopneumonia	+	HAdV-7	cell swallon	-	1:32	1:128
5	female	10m	03/02/2009	bronchopneumonia	+	/	-	-	<1:8	/^d^
6	male	4m	05/02/2009	bronchopneumonia	+	HAdV-7	Cell swallon	+/-	<1:8	/^d^
7	female	1y	07/02/2009	bronchopneumonia	+	/	ND	-	1:32	/^d^
8	male	10m	28/01/2009	congenital cardiopathy	+^a^	/	-	+	1:128	/^d^
9	male	9m	31/01/2009	bronchopneumonia	+	HAdV-7	-	-	<1:8	/^d^
10	female	5m	18/01/2009	bronchopneumonia	-^c^	/	-	-	<1:8	/^d^
11	female	1y	27/01/2009	bronchopneumonia	+	HAdV-7	-	-	1:32	/^d^
12	female	8y	27/01/2009	bronchopneumonia	-^b^	/	-	-	1:8	/^d^
13	female	1y	22/01/2009	bronchopneumonia	+	HAdV-7	-	-	1:32	/^d^
14	female	9y	31/01/2009	bronchopneumonia	+	/	-	-	<1:8	1:8
15	male	1y	23/01/2009	bronchopneumonia	+^a^	HAdV-7	-	+	1:8	1:128
16	male	2y	23/01/2009	bronchopneumonia	+	/	cell lysis	+/-	<1:8	1:128
17	male	2y	22/01/2009	bronchopneumonia	+	HAdV-3	-	+	1:8	1:32
18	male	8m	28/01/2009	bronchopneumonia	-^b^	/	-	+	1:512	1:128
19	female	1y	09/02/2009	bronchopneumonia	+	HAdV-7	-	+/-	1:8	1:512
20	male	2y	09/02/2009	myocardial damage	-	/	cell lysis	-	1:8	1:32
21	male	2y	29/01/2009	bronchopneumonia	+	HAdV-7	-	-	1:8	1:512

a Positive for adenovirus and human respiratory syncytial virus.

b Positive for human Parainfluenza virus.

c Positive for rhinovirus.

d Convalescence sera unaviable because the patient did not return.

The sequence data were stored as standard chromatogram format files (.ab1) and were analyzed using Sequencer soft ware (version 4.0.5; Gene Codes, Ann Arbor, MI). The nucleotide sequence homology was inferred from the identity scores obtained using the BLASTn program (National Center for Biotechnology Information, Bethesda, MD). Sequence alignments were created with BioEdit Sequence alignment editor software (version 5.0.9; Tom Hall, North Carolina State University)[21]; and a phylogenetic dendrogram was constructed using the neighbor-joining method with the MEGA program (Sudhir Kumar, Arizona State University); and the reliability of the tree was estimated with 1,000 bootstrap pseudo-replicates[22].

### Nucleotide sequence accession numbers

The nucleotide sequence of the entire hexon gene for strain HAdV7-HZ/SHX/CHN/2009 determined in this study was deposited in the GenBank nucleotide sequence database under accession number GU230898.

## Competing interests

The authors declare that they have no competing interests.

## Authors' contributions

LT and XT performed the experiment. LW had made substantial contributions to acquisition of epidemiological information. LT drafted the manuscript. WX revised the manuscript. All authors read and approved the final manuscript.
